# Therapeutic potential of triptolide in autoimmune diseases and strategies to reduce its toxicity

**DOI:** 10.1186/s13020-021-00525-z

**Published:** 2021-11-07

**Authors:** Yaxin Cheng, Yonghua Zhao, Ying Zheng

**Affiliations:** 1 State Key Laboratory of Quality Research in Chinese Medicine, Institute of Chinese Medical Sciences, University of Macau, Macau, China; 2 Guangdong-Hong Kong-Macau Joint Lab on Chinese Medicine and Immune Disease Research, University of Macau, Macau, China

**Keywords:** Triptolide, Autoimmune diseases, Toxicity, Pharmacology, Pharmacodynamics

## Abstract

With the increasing epidemiology of autoimmune disease worldwide, there is an urgent need for effective drugs with low cost in clinical treatment. Triptolide, the most potent bioactive compound from traditional Chinese herb Tripterygium Wilfordii Hook F, possesses immunosuppression and anti-inflammatory activity. It is a potential drug for the treatment of various autoimmune diseases, but its clinical application is still restricted due to severe toxicity. In this review, the pharmacodynamic effects and pharmacological mechanisms of triptolide in autoimmune diseases are summarized. Triptolide exerts therapeutic effect by regulating the function of immune cells and the expression of cytokines through inflammatory signaling pathways, as well as maintaining redox balance and gut microbiota homeostasis. Meanwhile, the research progress on toxicity of triptolide to liver, kidney, reproductive system, heart, spleen, lung and gastrointestinal tract has been systematically reviewed. In vivo experiments on different animals and clinical trials demonstrate the dose- and time- dependent toxicity of triptolide through different administration routes. Furthermore, we focus on the strategies to reduce toxicity of triptolide, including chemical structural modification, novel drug delivery systems, and combination pharmacotherapy. This review aims to reveal the potential therapeutic prospect and limitations of triptolide in treating autoimmune diseases, thus providing guiding suggestions for further study and promoting its clinical translation.

## Introduction

In human body, there is an immune network composed of immune organs (bone marrow, thymus, spleen, etc.), immune cells (lymphocytes, mononuclear phagocytes, neutrophils, etc.), and immunoactive molecules (antibodies, cytokines, complements, etc.), which can protect the body from harmful or foreign substances such as microorganisms, parasites, and cancer cells. When the immune system mistakenly regards substances belonging to “self” as “non self”, normal cells will be attacked by autoantibodies and abnormally activated immune cells, resulting in autoimmune disease [1]. There are more than 80 autoimmune diseases, which can be classified into two general types: systemic autoimmune diseases, such as rheumatoid arthritis, ankylosing spondylitis, systemic lupus erythematosus, and psoriasis; organ-specific autoimmune diseases, such as inflammatory bowel disease and multiple sclerosis. Incidence and prevalence of autoimmune disease are increasing significantly worldwide, which account for 19.1% and 12.5% respectively at present [2]. Traditional clinical therapeutic methods of autoimmune disease include corticosteroids, nonspecific immunosuppressive agents (such as cyclophosphamide, cyclosporine, methotrexate and dimethyl fumarate), and biologic drugs (such as etanercept, infliximab, adalimumab and tocilizumab) [3]. Corticosteroids help to rapidly relieve inflammation and alleviate overactive immune response, but have many side effects in long-term use. Nonspecific immunosuppressive agents not only inhibit autoimmune response, but also suppress the body’s resistance to foreign invasion, thus increasing the risk of infections and malignant tumors. With the gradual clarification of the pharmacological mechanisms on these autoimmune diseases, targeted biotherapy for cytokines and their receptors have been developed. These biologic drugs contribute to maintaining effective remission in long-term treatment at a high cost, while also increase the risk of infections [4].

**Fig. 1:**
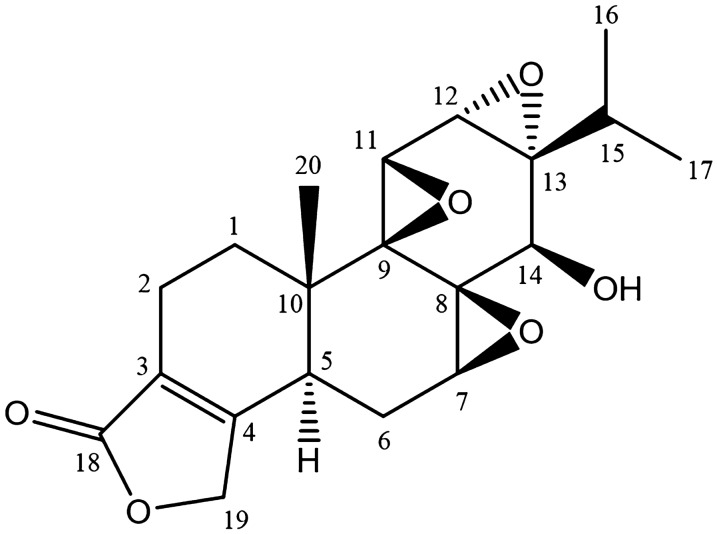
Chemical structure of triptolide

Traditional Chinese herb *Tripterygium Wilfordii* Hook F (TWHF) is a woody vine of the Celastraceae family, which mainly distributes in Eastern and Southern China, Korea and Japan [5]. The first application of this Chinese materia medica can be traced to more than two thousand years ago, when it was used to treat edema, fever, chills, sores, carbuncle and arthralgia [6]. However, western medicine did not realize the potential value of TWHF until researchers discovered the effectiveness of Tripterygium extracts in the therapy of patients with rheumatoid arthritis [7]. Since then, TWHF has been clinically used to treat various inflammatory diseases and autoimmune disorders owing to its cost-effectiveness. Evidence indicate TWHF and its extracts could exert a regulatory effect on immune function by regulating the proliferation and activation of T and B cells, proportion of T cell subsets, inflammatory response of monocyte and macrophage, production of immunoglobulin and cytokines [8]. Initially, water extracts from roots and leaves of TWHF were mostly used in clinical treatment. Due to the frequent occurrence of adverse reactions, ethyl acetate extract and chloroform-methanol extract were developed to reduce toxicity [9]. More than 70 ingredients have been isolated from TWHF, and the number is continuously increasing with study progress [4]. Triptolide (C_20_H_24_O_6_, Fig. 1) is the most potent bioactive compound from TWHF, whose action had been demonstrated to possesses immunosuppression, anti-inflammatory and anti-tumor activity [10]. However, due to severe hepatic, nephric, heart and gastrointestinal toxicity, the clinical application of triptolide is seriously restricted. The C-14β-hydroxyl group and the lactone ring of triptolide molecule are crucial for its efficacy and also for cytotoxicity [11]. Therefore, appropriate structural modification and suitable delivery system, which could maintain the bioactivity of triptolide but reduce its systemic toxicity, are required to be developed for clinic therapy.

In order to comprehensively and deeply understand of pharmacodynamic effects and pharmacological mechanisms of triptolide in various autoimmune diseases, we summarized and reviewed the relevant research progress. Additionally, we also pay great attention to the strategies to reduce toxicity of triptolide, aiming to promote the clinical translation and provide guiding suggestions for further research.

## Efficacy of triptolide in autoimmune diseases

**Fig. 2:**
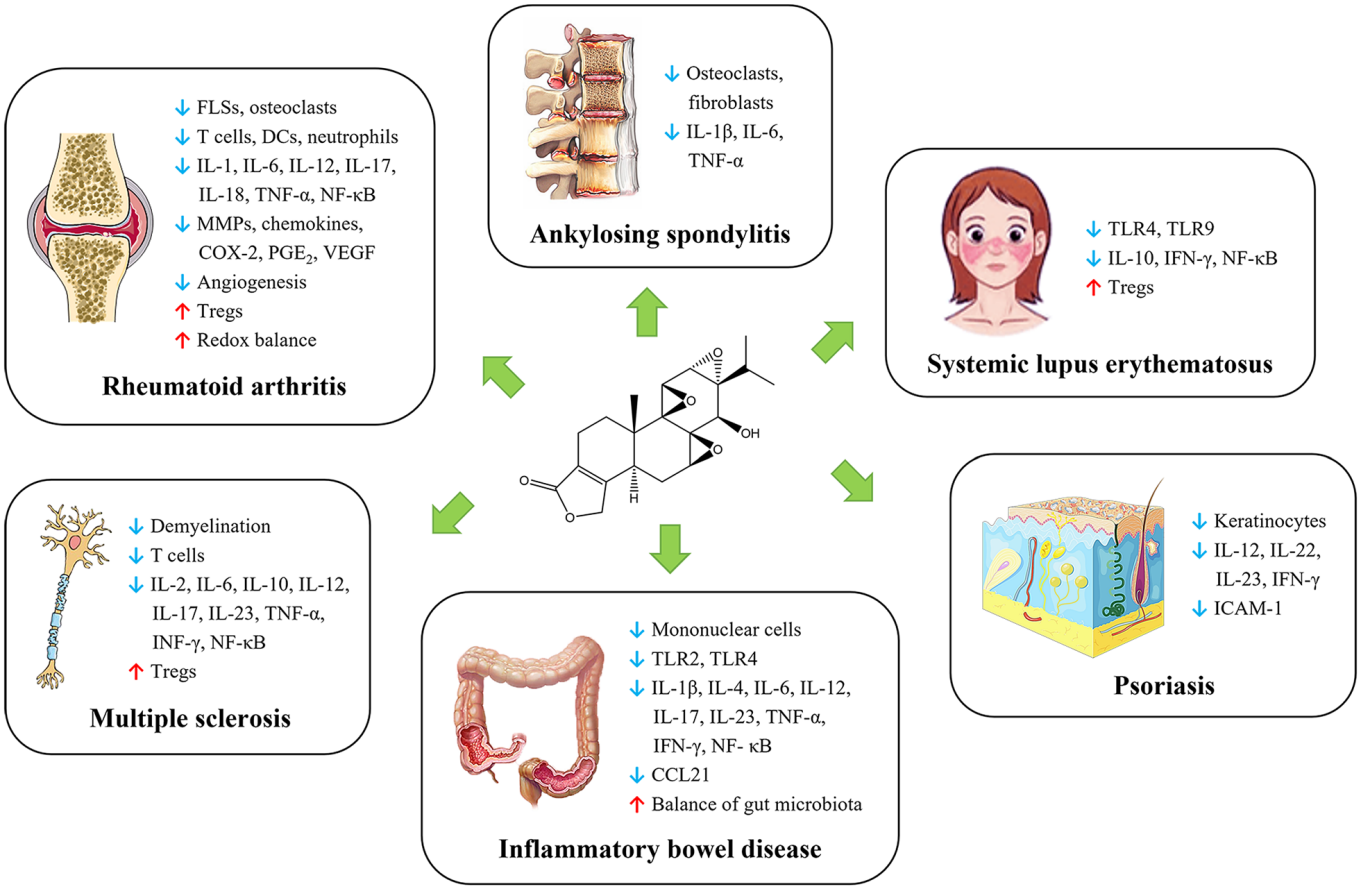
Schematic illustration of triptolide in the treatment of autoimmune diseases

The therapeutic potential of triptolide has been studied in various autoimmune diseases summarized in Fig. 2, including rheumatoid arthritis, ankylosing spondylitis, systemic lupus erythematosus, psoriasis, inflammatory bowel disease, and multiple sclerosis.

### Rheumatoid arthritis

**Fig. 3:**
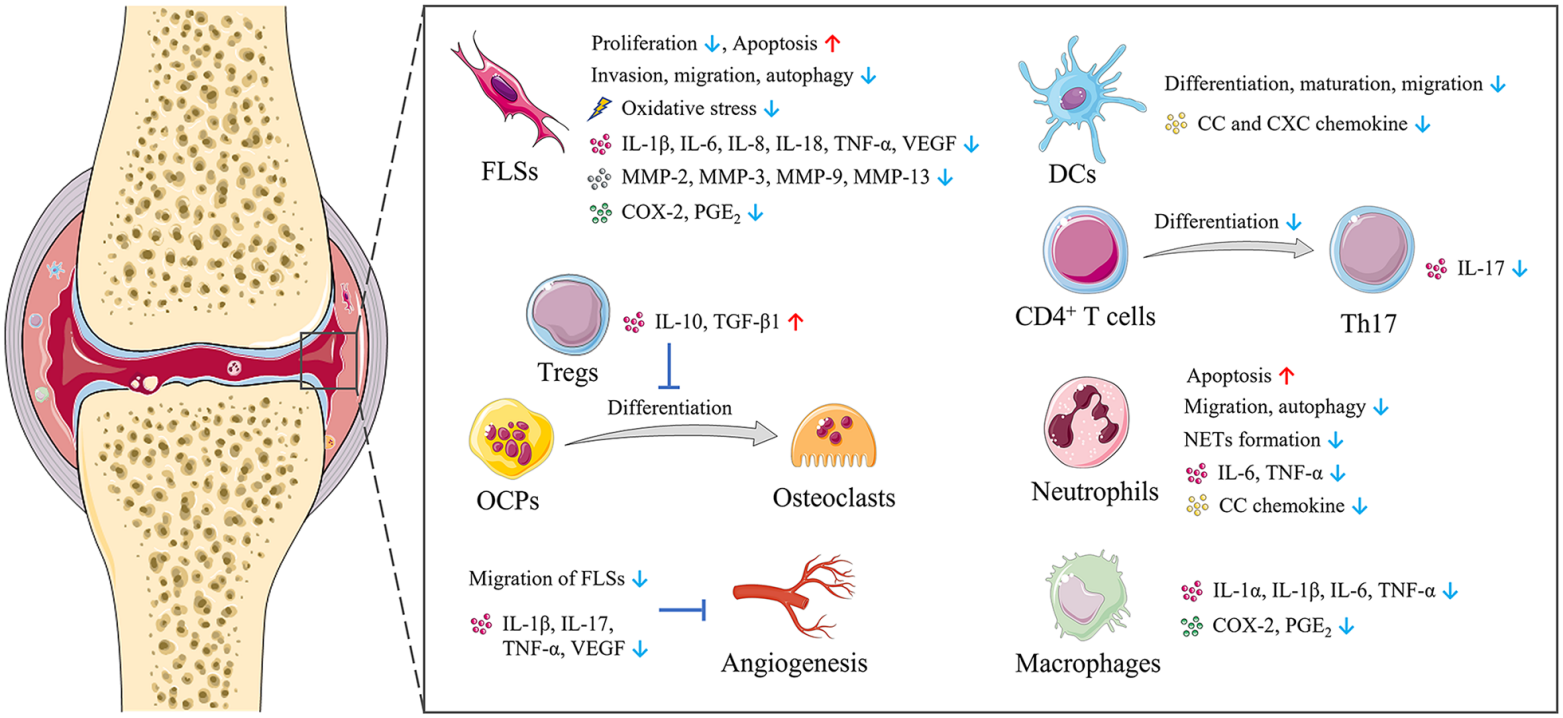
Triptolide plays a therapeutic role in RA by regulating the function of FLSs, immune cells, and osteoclasts, as well as the progress of angiogenesis

Rheumatoid arthritis (RA) is a chronic autoimmune disorder characterized by autoantibody production, proliferation of synoviocytes, infiltration of immune cells, joint destruction, and angiogenesis, which affects about 1% of the population [12]. Triptolide plays a therapeutic role in RA by regulating the function of fibroblast-like synoviocytes (FLSs), immune cells, and osteoclasts, as well as the progress of angiogenesis (Fig. 3).

#### Regulation of FLSs

Previous researches have proved that FLSs appear to be one of the most important factors in the pathogenesis of RA, which has a great contribution to the synovial inflammation and cartilage destruction [13, 14]. After activated, FLSs can hyperproliferate, flee apoptosis, invade the unaffected bone and joint, just like tumor cells [15]. Therefore, FLSs are considered as a potential therapeutic target for blocking the pathogenesis of RA. Tong et al. [16] demonstrated for the first time that triptolide treatment can directly inhibit the proliferation of FLSs. Su et al. [17] found the anti-proliferative effect is attributed to the fact that triptolide treatment can arrest the cell cycle and induced cell apoptosis of FLSs. Notably, they used high-resolution atomic force microscopy to visually determine the effects of triptolide on FLSs for the first time. The results showed that triptolide destroyed the morphology of FLSs, which is specifically manifested in increased roughness and stiffness. Additionally, Yang et al. [18] observed that triptolide administration significantly inhibited the invasion and migration of FLSs by blocking the Jun N-terminal kinase (JNK)/mitogen-activated protein kinase (MAPK) signal pathway, thus relieved arthritic symptoms and bone destruction in collagen-induced arthritis (CIA) mice model. Furthermore, Xie et al. [19] demonstrated that triptolide treatment suppressed cell mobility and maintained redox balance through inhibiting autophagy by activating phosphatidylinositol 3‑kinase (PI3K)/protein kinase B (AKT) signaling pathway in FLSs. Besides, Lu et al. [20] reported that triptolide treatment can effectively inhibit the expression of interleukin (IL)-18 and its receptor in FLSs, as well as suppress nuclear factor kappa B (NF-κB) activity.

#### Inhibition of bone resorption and cartilage degradation

Bone destruction in inflammatory joints is the most typical characteristic of RA, which is caused by the activation of osteoclasts. When a large number of osteoclasts differentiate from osteoclast precursors (OCPs), the bone resorption process will be enhanced and lead to the destruction of bones and joints [21]. Bone resorption mediated by osteoclast is regulated by the receptor activator of nuclear factor-κB (RANK) ligand (RANKL) and osteoprotegerin (OPG) [22]. RANKL can stimulate osteoclastic formation and differentiation when binding with RANK, and OPG can prevent bone resorption by competing with RANKL for RANK [23]. Liu et al. [24] found that triptolide treatment significantly down-regulated the expression of RANKL and RANK, and up-regulated the expression of OPG in CIA mice, thereby inhibiting osteoclast formation in the bone destruction area. However, the chronic and recurrent symptoms of patients with RA are not completely consistent with CIA or adjuvant-induced arthritis (AIA) mouse models, but more similar to the spontaneously forming RA models in tumor necrosis factor-transgenic (TNF-Tg) mice. Wang et al. [25, 26] reported triptolide treatment could effectively alleviate the deformation and swelling in the joints of TNF-Tg mice by promoting the apoptosis of OCPs, inhibiting osteoclast formation and bone resorption. Matrix metalloproteinases (MMPs), the major proteases participated in the invasion and degradation of cartilage extracellular matrix, are significantly overexpressed in the synovial fluid of patients with RA [27]. Triptolide can suppress the expression of MMPs, such as MMP-1, MMP-2, MMP-3, MMP-9 and MMP-13, as well as augment the expression of tissue inhibitors of MMPs in FLSs [19, 28, 29]. In addition, Lin et al. [30] demonstrated triptolide treatment blocked cartilage degradation in CIA mice by reducing the levels of IL-1, IL-6, TNF-α, cyclooxygenase (COX)-2 and prostaglandin E2 (PGE_2_) in FLSs and macrophages, which can induce the production of MMPs in inflamed joints.

#### Regulation of immune cells

T lymphocytes play a critical role in immune regulation in RA, especially CD4^+^ T cells, which are necessary for disease initiation and development [31]. When stimulated by various cytokines, CD4^+^ T cells will differentiate into subsets of cells with different effector functions, including Th1, Th2 and Th17 cells [32]. Zhou et al. [33] demonstrated triptolide treatment could induce immunosuppress response by reducing CD4^+^ T cells in periphery, subsequently improving arthritis symptom and delaying the onset of RA in CIA mice. Moreover, Wang et al. [34] reported that triptolide treatment inhibited Th17 differentiation and IL-17 expression by downregulating IL‐6‐induced signal transducer and activator of transcription 3 (STAT3) phosphorylation, which is a cytokine‐activated key regulator in signaling molecules involved in Th17 development. Autoreactive T cells can be induced and activated by overexpression of T cell receptor (TCR) variable gene fragments. Wang et al. [35] found triptolide treatment inhibited the expression of TCR BV15 and TCR BV19, which was conducive to the therapeutic effect of triptolide in RA. In addition to Th1 and Th17 cells, CD4^+^CD25^+^Foxp3^+^ T cells, also named regulatory T cells (Tregs), is also one of key T cell subsets in RA pathogenesis by limiting autoimmune responses [36]. Xu et al. [37] proved that Tregs could reduce osteoclast differentiation and bone resorption, while triptolide treatment can enhance the suppressive effects of Tregs by increasing the secretion of IL-10 and transforming growth factor (TGF)-β1.

Dendritic cells (DCs), the most potent professional antigen-presenting cell, initiate immunity in RA by activating T cells and eliciting inflammatory processes involved in RANK/RANKL pathway [38]. Chen et al. [39] observed that triptolide treatment inhibited the differentiation, maturation, allostimulation and migration of human monocyte-derived DCs in a nontoxic concentration. Besides, triptolide treatment can reduce cysteine-cysteine (CC) and cysteine-X-cysteine (CXC) chemokines production by blocking Janus kinase (JAK)/STAT3 signaling pathway and NF-κB activation, thus inhibiting the infiltration of neutrophils and T cells induced by DC [40]. The attraction of immunity cells into the inflammatory joints is controlled by the interaction between chemokines and chemokine receptors on the leukocyte surface. CC chemokine receptor 5 (CCR5), the receptor for CC chemokine ligand 5 (CCL5), macrophage inflammatory protein (MIP)-1α, MIP-1β, and monocyte chemotactic protein (MCP)-2, show strong expressions on FLSs, T cells, and DCs in RA [41]. Wang et al. [42, 43] discovered that triptolide treatment significantly inhibited the expressions of CCL5, MIP-1α, MCP-1 and CCR5 in AIA mice, which is conducive to the therapeutic effects of triptolide on RA.

Neutrophils, as an essential part of innate immune system, normally circulate in the bloodstream and migrate to the site of inflammation in response to inflammatory stimuli [32]. Neutrophils have recently been considered to play an active role in orchestrating the progression of RA by secreting cytokines and releasing neutrophil extracellular traps (NETs) [44, 45]. Huang et al. [46] found triptolide treatment ameliorated arthritis in AIA mice by inducing neutrophil apoptosis and reducing neutrophil infiltration, as well as inhibiting neutrophil autophagy and NET formation. They also determined that triptolide treatment suppressed the expression and secretion of cytokines and chemokines in activated neutrophils, including IL-6, TNF-α, granulocyte-macrophage colony-stimulating factor (GM-CSF), and CCL5, which contributes to the recruitment of discrete innate and adaptive immunity cells [47].

#### Inhibition of angiogenesis

Angiogenesis, as a vital factor in maintaining inflammation and immune responses, has been suggested as a potential therapeutic target for RA [48]. Kong et al. [49] found that triptolide exerted antiangiogenic effect in CIA mice by downregulating the expression of angiogenic activators such as IL-17, TNF-α and vascular endothelial growth factor (VEGF), as well as suppressing the IL-1β-induced activation of MAPK signal pathway.

### Ankylosing spondylitis

Ankylosing spondylitis (AS) is a chronic rheumatic disease affecting sacroiliac joints and spine with a prevalence of 0.1–1.4% [50]. AS frequently occurs in young people around 26 years old and is more prevalent in males than in females with a ratio of roughly 2–3 to 1 [47].

**Fig. 4:**
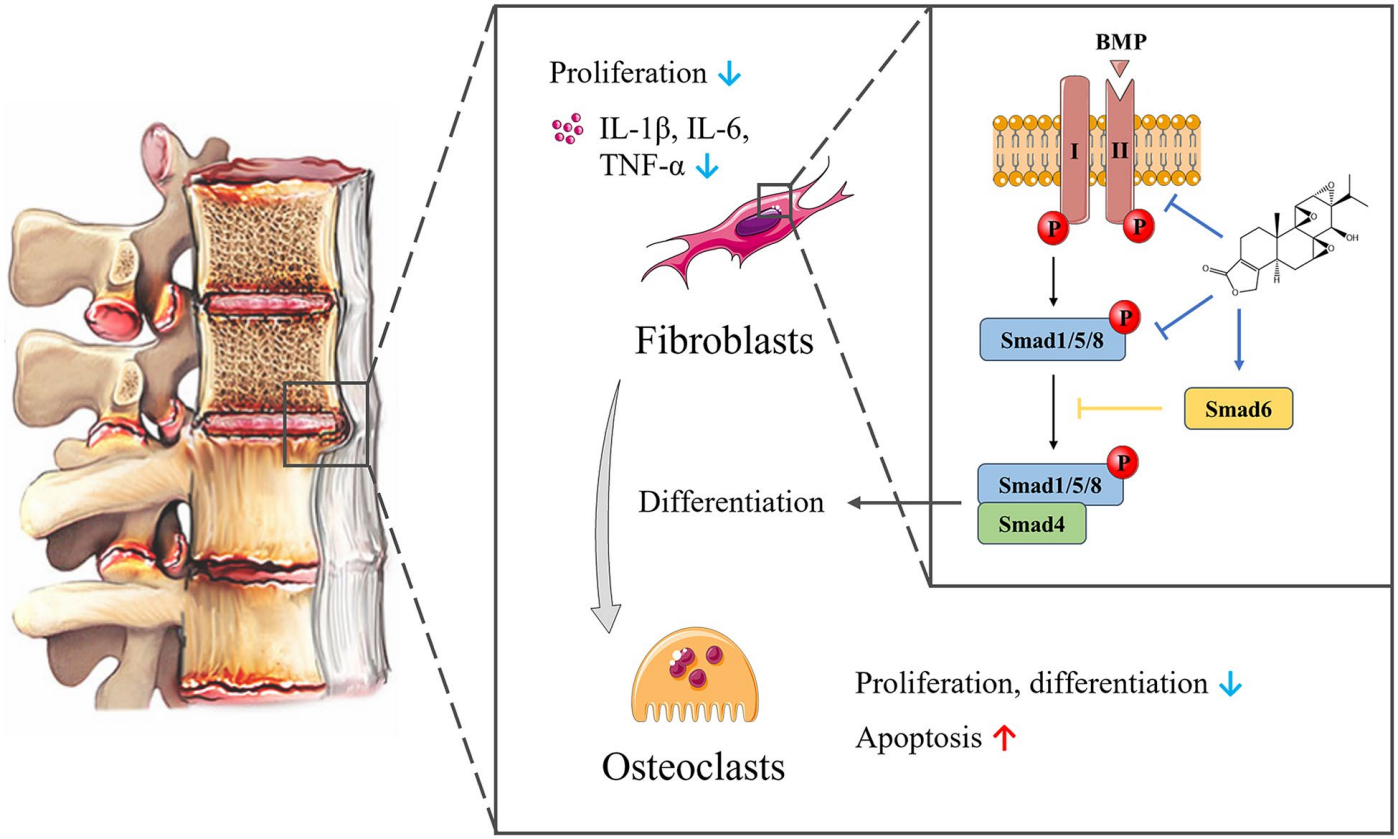
Triptolide treatment can inhibit bone formation by regulating the proliferation, differentiation and secretion of AS fibroblasts and osteoblasts

AS is characterized by the formation of bony spurs and associated functional impairment, ultimately leading to deformity. Therefore, prevention of excessive bone formation is a key therapeutic goal in the treatment of AS [51]. Triptolide treatment can inhibit bone formation by regulating the proliferation, differentiation and secretion of AS fibroblasts and osteoblasts (Fig. 4). Ji et al. [52] demonstrated that triptolide treatment inhibited proliferation of MC3T3-E1 mouse osteoblast cells, as well as induced cell cycle arrest and apoptosis. Additionally, treatment with triptolide suppressed collagen formation, alkaline phosphatase activity and calcium deposition, suggesting an inhibitory effect of triptolide on osteoblast differentiation. Bone morphogenetic proteins (BMPs) play a critical role in osteoblast differentiation and transmit signals through Smad proteins upon specific binding to target surface receptors on mesenchymal cells [53]. Wang et al. [54] suggested that triptolide treatment can inhibit the proliferation and osteogenic differentiation of AS fibroblasts, and reduce the expression of TNF‑α, IL‑1β and IL‑6 through the BMP/Smad pathway.

It appears that hereditary genes play an essential significant role in the pathogenesis of AS. More than 90% of patients with AS have a particular marker that can be found on their white platelets, called human leukocyte antigen (HLA)-B27 [55]. A study screened 12,264 chemicals by using high-throughput technology to explore their effects on HLA-B27 gene promoter, and discovered two HLA-B27 suppressors (celastrol and pristimerin) derived from TWHF [56]. Triptolide were one of these screened chemicals, but its detailed effect on HLA-B27 was not shown in this study.

### Systemic lupus erythematosus

Systemic lupus erythematosus (SLE) is a systemic autoimmune disease characterized by antinuclear antibody and multi-system involvement, which is more common in women of child-bearing age [57].

Lupus-prone MRL/lpr mice are one of the most attractive animal models to evaluate effective therapies of SLE, due to their spontaneity, multiple tissue destruction and sexual dimorphism [58]. Huang et al. [59] demonstrated that triptolide treatment alleviated skin damage and renal histopathologic characteristic symptoms in MRL/lpr mice, meanwhile downregulated the expressions of Toll-like receptor (TLR) 4, TLR9, IL-10, interferon gamma (IFN-γ) and NF-κB. MiR-125a-5p has been shown to be a key regulator for stabilizing Treg-mediated immune homeostasis and consequently controls autoimmunity and inflammation [60]. Zhao et al. [61] reported that triptolide treatment elevated Treg number via upregulating miR-125a-5p in spleen tissues of MRL/lpr mice, indicating that triptolide might have the potential therapeutic utility for the treatment of SLE.

### Psoriasis

**Fig. 5:**
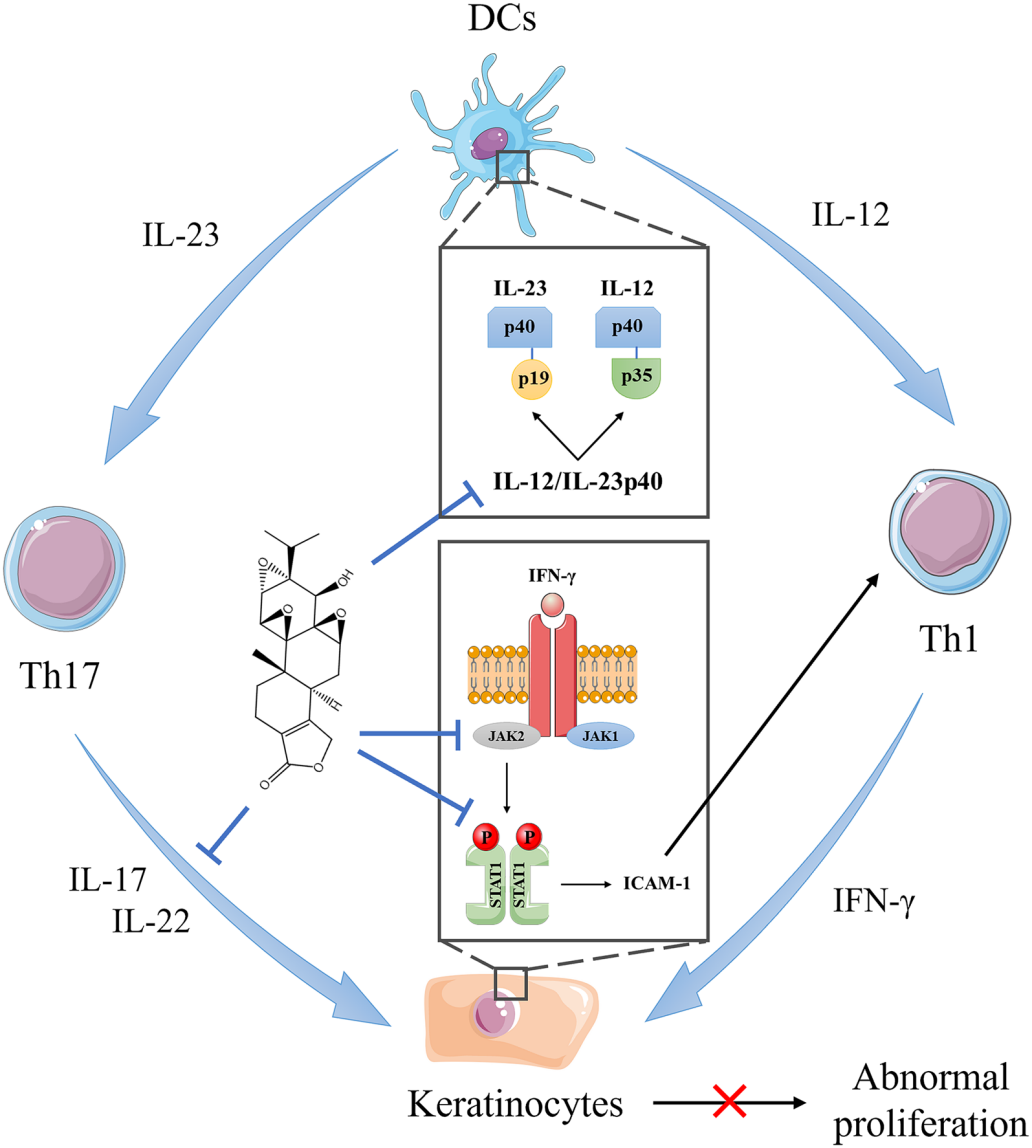
Triptolide treatment can suppress keratinocytes proliferation and immune infiltration by regulating APC function and blocking IFN-γ signaling

Psoriasis is a chronic, disfiguring, autoimmune skin inflammatory disease, which affects 2–3% population worldwide [62]. The main characteristics of psoriasis are scaly erythematous plaques, abnormal proliferation of keratinocytes and recruitment of immune cells into the skin [63]. Triptolide treatment can suppress keratinocytes proliferation and immune infiltration by regulating APC function and blocking IFN-γ signaling (Fig. 5).

Although the molecular mechanism of psoriasis has not been thoroughly elucidated, many studies indicate that IL-23/IL-17 axis makes a significant contribution to psoriasis pathogenesis [64]. IL-12 and IL-23, which are closely related heterodimeric cytokines produced by DCs, share the common subunit p40. Under the stimulation of IL-23, which promotes the differentiation and migration of autoimmune Th17 cells, the latter can recognize epidermal autoantigens and secrete IL-17. Zhang et al. [65] demonstrated triptolide treatment inhibited the expression of IL-12/IL-23p40 by activating CCAAT/enhancer-binding protein-α in DCs, providing a strong enlightenment for the therapeutic application of triptolide in psoriasis.

The abnormal proliferation and differentiation of keratinocytes are related to pro-inflammatory cytokines, such as IFN-γ and IL-22. Tu et al. [66] found that triptolide treatment suppressed the expression of IFN-γ receptor α and blocked IFN‐γ signal transduction in keratinocytes via JAK2/STAT1 Pathway. He et al. [67] proved that triptolide treatment can reverse the uncontrolled proliferation and poor differentiation of keratinocytes induced by IL-22 through upregulating miR-181b-5p.

### Inflammatory bowel disease

Inflammatory bowel disease (IBD) is a series of inflammatory conditions of colon and small intestine resulting from dysregulation of mucosal immune response, comprised of ulcerative colitis (UC) and Crohn’s disease (CD) [3].

**Fig. 6:**
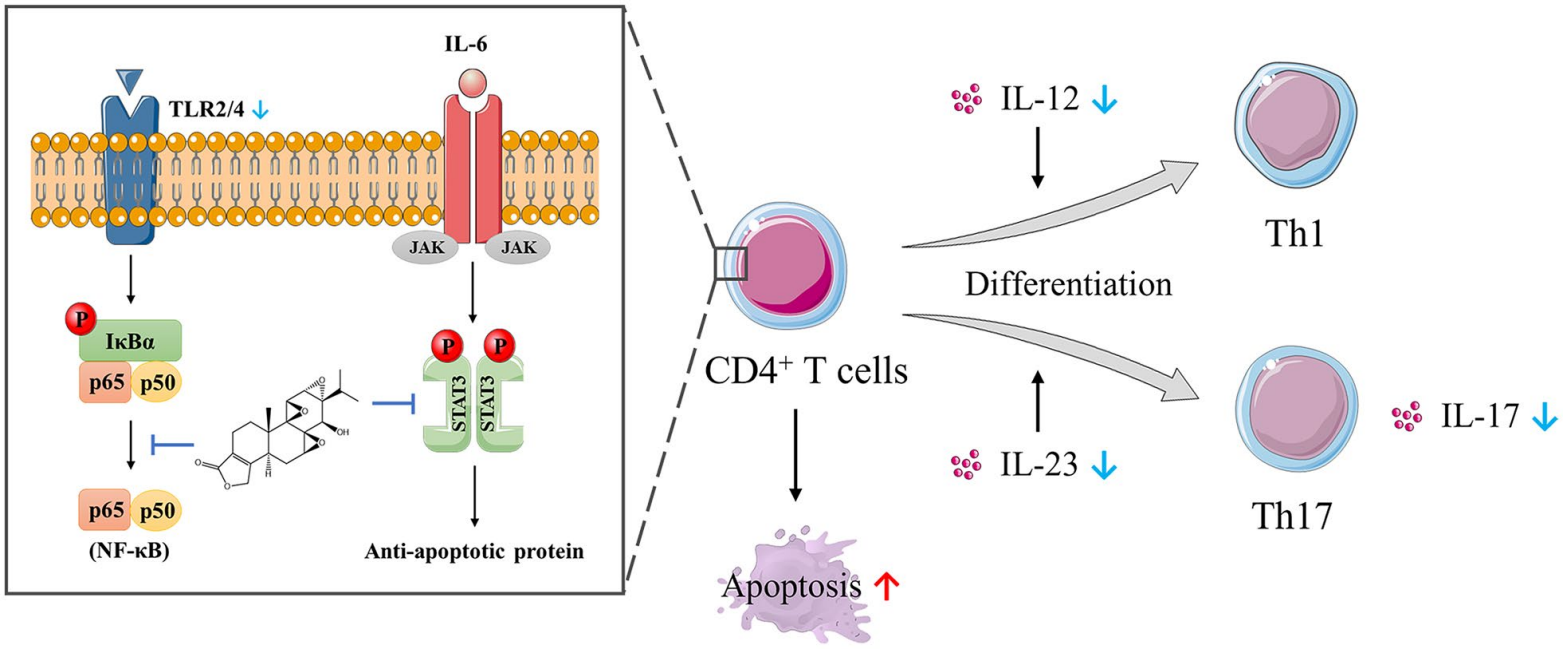
Triptolide treatment can alleviate colitis by regulating Th1 and Th17 pathway

CD is characterized by increased intestinal permeability caused by defective intestinal tight junction (TJ) barrier, which allows paracellular infiltration of luminal antigens and promotes intestinal inflammation [68]. The elevated level of TNF-α has been proposed as a central factor contributing to the alteration of intestinal TJ permeability in CD patients. Thomas et al. [69] indicated that triptolide treatment prevented TNF-α-induced decrease in zonula occludens-1 protein expression in vitro via NF-κB pathways, which is a peripheral membrane phosphoprotein playing a regulatory effect in the modulation of TJ barrier. IL-10 deficient (IL-10^−/−^) mice, a typical experimental model of IBD, spontaneously develop chronic colitis mediated by Th1 and Th17 cell with many similarities to human CD [70]. Research shows that triptolide treatment can alleviate colitis by regulating Th1 and Th17 pathway (Fig. 6). Wei et al. [71] observed that administration of triptolide ameliorated the wasting syndrome and inflammation-mediated injury in colon of IL-10^−/−^ mice, as well as reduced the expressions of TNF-α, IFN-γ, IL-4, IL-12 and IL-23 in colon mucosa through inhibiting NF-κB activation. Furthermore, Yu et al. [72] demonstrated that triptolide treatment inhibited NF-κB activation by suppressing TLR signaling pathway and the expressions of TLR2 and TLR4. Additionally, Li et al. [73, 74] proved that triptolide treatment controlled the development of colitis in IL-10^−/−^ mice and cultured colonic explants from CD patients, which was attributable to suppression of IL-6/STAT3 pathway, reduced expression of IL-17 and induced apoptosis of lamina propria mononuclear cells.

The interaction between host immune system and indigenous gut microbiota is related to the pathogenesis of IBD [75]. Once gut microbiota is loss of homeostasis, the immune and metabolic functions of gastrointestinal tract may be affected, leading to inflammatory response and host cell damage. In rectal mucosa of patients with UC, the population of pathogenic bacteria such as Escherichia coli, Peptostreptococcus and Bacteroides fragilis are increased significantly, while the population of protective bacteria such as Lactobacillus and Bifidobacterium are decreased [76]. Wu et al. [77] reported the biological diversity and composition of gut microbiota recovered after administration of triptolide, thus inhibiting the expressions of IL-6, TNF-α, and IL-17 and improving the symptom of 4,4-dimethyl-4-silapentane-1-sulfonic acid (DSS)-induced UC mice. Their results also showed that symptoms of UC could be alleviated by transplanting feces from mice treated with triptolide. Besides, Zhang et al. [78, 79] found triptolide treatment exerted good therapeutic effect on DSS-induced UC mice by decreasing the expressions of CCL21 and IL‑1β.

### Multiple sclerosis

Multiple sclerosis (MS) is a demyelinating autoimmune disease of central nervous system (CNS), characterized by perivascular lymphocytes infiltration and disseminated demyelinating lesions [80].

**Fig. 7:**
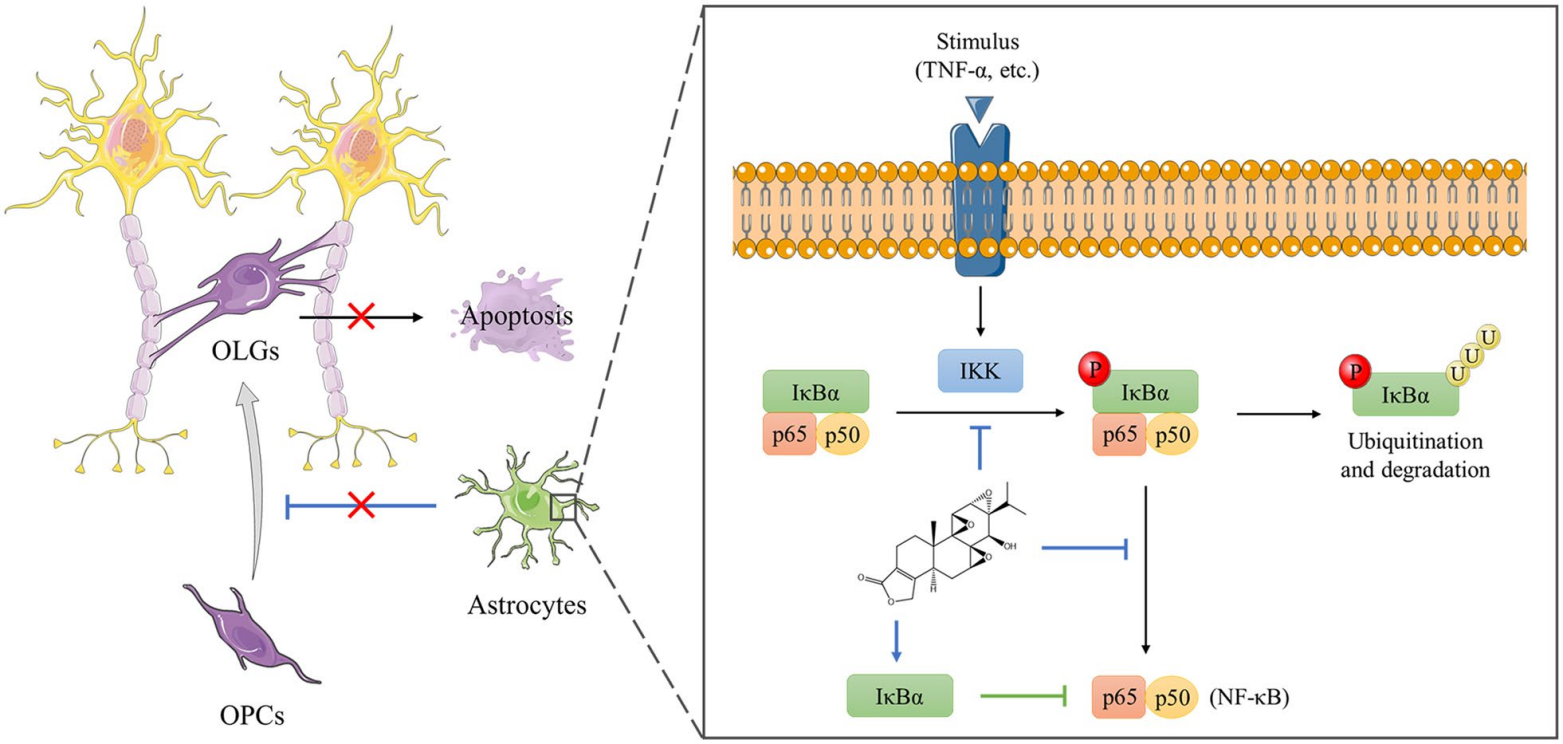
Triptolide treatment can protect OLGs from toxic demyelination by suppressing NF-κB pathway

Experimental autoimmune encephalomyelitis (EAE) is an animal model similar to human MS, which can be induced by immunization with myelin components or transference of encephalitogenic T cells. Kizelsztein et al. [81] demonstrated that triptolide treatment significantly reduced inflammatory infiltration and demyelination in the CNS of EAE mice through suppressing NF-κB pathway. In addition, Wang et al. [82] found that triptolide treatment up-regulated Foxp3 expression in EAE mice, which is an important marker of Treg cells. Compared with EAE modle, toxin-induced demyelinating models like the cuprizone model can selectively investigate the molecular factors contributing to remyelination process by recruiting or activating oligodendrocyte precursor cells (OPCs) and preventing apoptosis of mature oligodendrocytes (OLGs) [83]. Using this model, Sanadgol et al. [84] revealed that treatment with low-dose of triptolide could suppress NF-κB pathway related to apoptosis and inflammatory markers and protect OLGs from toxic demyelination, thus leading to increase in OPCs population (Fig. 7).

## Toxicity of triptolide

### Hepatotoxicity

The molecular mechanism of triptolide toxicity is considered to be related to its epoxide structure, which may covalently bound to biological macromolecules in the body as deficient electron donor and subsequently breaks these molecules [85]. Animal studies have shown that triptolide administration can induce damage in multiple organs and tissues, and even lead to the death (Table 1). The liver is one of the most significant organs involved in triptolide-induced toxicities. Wang et al. [86] observed obvious focal necrosis with inflammatory cell infiltration in hepatocytes of female Wistar rats treated with 1 mg/kg triptolide by oral gavage. Meanwhile, the serum concentrations of alanine transaminase (ALT), aspartate transaminase (AST) and γ-glutamyl transpeptidase, as key biochemical parameters of liver injury, were increased compared with the control group. Li et al. [87] showed that serum levels of ALT and AST in male BALB/C mice increased to 9.1 and 9.8 times respectively after intraperitoneal injection of 1.0 mg/kg triptolide. They also found that triptolide treatment induced oxidative stress injury in HepG2 cells, and its cytotoxicity could be exacerbated by knockdown of nuclear factor E2-related factor 2 (Nrf2) or be relieved by overexpression of Nrf2, which was a leading factor in cells for the protection against oxidative stress. In addition, Fu et al. [88] indicated that triptolide administration induced liver injury accompanied by microcystic steatosis, hyperlactatemia and increased oxidative stress, resulting from the inhibition of mitochondrial respiratory chain and secondary β-oxidative damage. Furthermore, Yuan et al. [89] suggested oxidative stress induced by triptolide might be related to the activation of NLR family pyrin domain containing 3 (NLRP3) inflammasome, which can result in the release of cytokines and recruitment of the inflammatory cells, and further augment the liver damage.

Besides, Wang et al. [90] showed that 0.5 mg/kg of triptolide enhanced the expansion of Th17 cells and suppressed the production of Tregs in female C57BL/6 mice, which may represent a new pathogenesis of TP-induced liver injury. Yang et al. [91] found that 0.4 mg/kg of triptolide increased serum total bile acid and inhibited hepatic gluconeogenesis in Wistar rats by inhibiting Sirt1/farnesoid X receptor (FXR) signaling pathway. Lu et al. [92] demonstrated that triptolide administration could induce hepatotoxicity by inhibiting the substrate affinity, activity and expressions of the cytochromes P450 (CYP450) isoforms 3 A, 2C9, 2C19, and 2E1. Moreover, Liu et al. [93] reported triptolide treatment displayed lower toxicity and higher metabolic rate in male SD rats, revealing the role of CYP3A in the gender differences of triptolide toxicity, which is the main metabolic isozyme in male rats.

In addition, Li et al. [94] established an in vitro/in silico method by physiologically based pharmacokinetics (PBPK) modeling, which could extrapolate in vitro cytotoxicity data to in vivo hepatotoxicity blood concentrations and convert AUC to corresponding oral doses. The maximum safe dose (5% hepatotoxic probability) of triptolide they predicted is close to the clinical recommendation.

### Nephrotoxicity

The nephrotoxicity of triptolide histologically appears as the separation of proximal convoluted tubular epithelial cells, which is caused by the destruction of intercellular junctions and changes of paracellular permeability in proximal tubules [95]. To evaluate the nephrotoxicity of triptolide, the levels of blood urea nitrogen (BUN) and creatinine (Cr) in serum are used as biochemical makers. Yang et al. [96] revealed that a single dose induced severe renal injury, such as brush border injury, tubular epithelial cells abscission, and tubular obstruction, as well as a remarkable upregulation of BUN and Cr levels. They also [97] indicated that triptolide treatment caused serious oxidative stress and renal structural damage after a single large dose intraperitoneal injection of 1 mg/kg triptolide in male SD rats, while vitamin C, an antioxidant, significantly ameliorated triptolide-induced injury of renal function.

The toxicity of drugs under pathological conditions is different from that under normal physiological conditions. It is necessary to evaluate drug toxicity by using pathological model, which is helpful to fully understand the toxicity mechanism of drugs. Shen et al. [98] demonstrated that the nephrotoxicity of triptolide was increased in CIA rats, which might be due to explosion of TNF-α in blood, resulting in upregulation of organic cation transporter 2 (Oct2) expression in kidney. Overexpressed Oct2 could transport excessive triptolide into the kidney, which aggravated the nephrotoxicity of triptolide after long-term administration.

### Reproductive toxicity

For male reproductive toxicity, significant changes were observed in seminiferous tubules and epididymides [99]. After treated with triptolide by oral administration for 8 weeks, the weights of testis and epididymis, sperm content and motility were decreased significantly in male SD rats. Ma et al. [100] suggested that the impairment of spermatogenesis may be caused by abnormal lipid and energy metabolism in testis via down-regulation of peroxisome proliferator-activated receptor (PPAR) mediated by triptolide. Li et al. [101] reported triptolide treatment decreased the expression of breast cancer resistance protein (an efflux transporter) in the testis, and further increased the testis content, which contributed to its cumulative testicular toxicity.

For female reproductive toxicity, long-term treatment with triptolide prolonged the estrous cycles, suppressed estrogen levels, reduced ovary and uterus weights in female SD rats [102]. Zhang et al. [103, 104] revealed that triptolide treatment inhibited the expression of estrogen and progesterone synthesis enzymes by disrupting cyclic adenosine monophosphate (cAMP)/protein kinase A (PKA) pathway, causing the decrease of estradiol and progesterone synthesis. Besides, granulosa cells apoptosis by endoplasmic reticulum stress pathway and antiapoptotic function impairment may partly mediate triptolide-induced ovary toxicity [105].

### Other toxicity

Except for hepatotoxicity, nephrotoxicity and reproductive toxicity, triptolide administration could also lead to damage in other organs, such as heart, spleen, lung, gastrointestinal tract, and skin. In an acute toxicity test, triptolide administration increased the heart/body ratio and caused myocardial fiber breakage, cardiomyocyte hypertrophy, increased cell gaps, and nuclear dissolution in treated male rats [106]. Zhou et al. [107] demonstrated triptolide administration induced cardiotoxicity via oxidative stress in male BALB/C mice treated with 1.2 mg/kg triptolide by intravenous injection, which was associated with down-regulated Nrf2 activation and reactive oxygen species (ROS)-mediated mitochondria-dependent apoptosis pathway. They also found the enhancement of autophagy by rapamycin (a known promoter of autophagy) could mediate the removal of dysfunctional mitochondria, modulate cytotoxicity by suppression of cell death in cardiomyocytes exposed to triptolide, and also attenuate triptolide-induced oxidative stress damage in heart tissue of BALB/C mice [108]. The relative weight of spleen in triptolide-treated female rats was about 2 times higher than normal, accompanied by dilation and hyperemia of splenic sinuses [93]. Shao et al. [109] observed spleen necrosis characterized by the destruction of splenic corpuscle, the collapse of lymphocyte and hyperplasia of macrophage in male rats treated with 1.2 mg/kg triptolide. Meanwhile, local abscess formation and bacterial colony were prominent within lung. The most common adverse reactions to triptolide occur in the gastrointestinal tract and skin, such as nausea, vomiting, bellyache, diarrhea, duodenal ulcer, gastrointestinal bleeding, skin rash, and changes in skin pigmentation. Oral administration of triptolide can easily lead to gastrointestinal toxicity, such as nausea, emesis, bellyache, diarrhea, duodenal ulcer, and gastrointestinal bleeding. Marked hyperemia and infiltration of inflammatory cells occurred in gastric mucosa of rats when given an oral dose of triptolide suspension at 1.0 mg/kg [110]. Skin irritation is common in topical application of triptolide. Chen et al. [111] reported that the aqueous solution containing 0.025% triptolide could induce obvious erythema and edema on skin of rabbits after transdermal administration for 7 days.

In addition, a clinical trial demonstrated that although triptolide treatment could significantly improve the condition of most patients with rheumatoid arthritis, triptolide induced considerable serious toxicity, including urinary abnormalities, myocardial damage, leukopenia, and increased ALT level, resulting in a 47% withdrawal rate [112].

**Table 1:** The systemic toxicity of triptolide

Toxicity	Animals	Administration route	Minimum toxic dose	References
Hepatotoxicity	Female Wistar rats	Oral gavage	1 mg/kg/day, 14 days	[86]
	Male BALB/C mice	Intraperitoneal injection	1 mg/kg, 24 h	[87]
	Female SD rats	Oral gavage	0.4 mg/kg/day, 28 days	[88, 93]
	Female C57BL/6 mice	Oral gavage	0.6 mg/kg, 24 h	[89]
	Female C57BL/6 mice	Oral gavage	0.5 mg/kg, 24 h	[90]
	Female Wistar rats	Oral gavage	0.4 mg/kg/day, 28 days	[91]
	Female SD rats	Oral gavage	0.2 mg/kg/day, 28 days	[92]
Nephrotoxicity	Female Wistar rats	Oral gavage	0.2 mg/kg/day, 28 days	[95]
	Male SD rats	Intraperitoneal injection	1 mg/kg, 48 h	[96, 97]
	Female Wistar rats	Oral gavage	0.5 mg/kg/day, 28 days	[98]
Reproductive toxicity	Male SD rats	Oral gavage	0.1 mg/kg/day, 56 days	[99]
	Male mice	Intraperitoneal injection	0.06 mg/kg/day, 14 days	[100]
	Male C57BL/6 mice	Oral gavage	0.125 mg/kg/day, 15 days	[101]
	Female NIH mice	Oral gavage	0.025 mg/kg/day, 50 days	[105]
Cardiotoxicity	Male rats	Oral gavage	0.1 mg/kg/day, 14 days	[106]
	Male BALB/C mice	Intravenous injection	1.2 mg/kg, 24 h	[107, 108]
Splenic toxicity	Female SD rats	Oral gavage	0.4 mg/kg/day, 28 days	[93]
	Male SD rats	Oral gavage	1.2 mg/kg, 24 h	[109]
Lung toxicity	Male SD rats	Oral gavage	1.2 mg/kg, 24 h	[109]
Gastrointestinal toxicity	SD rats	Oral gavage	1.0 mg/kg, 1 h	[110]
Skin irritation	New Zealand rabbits	Transdermal administration	0.025 %, 7 days	[111]

## Strategies to reduce toxicity of triptolide

### Chemical structure modification

**Fig. 8:**
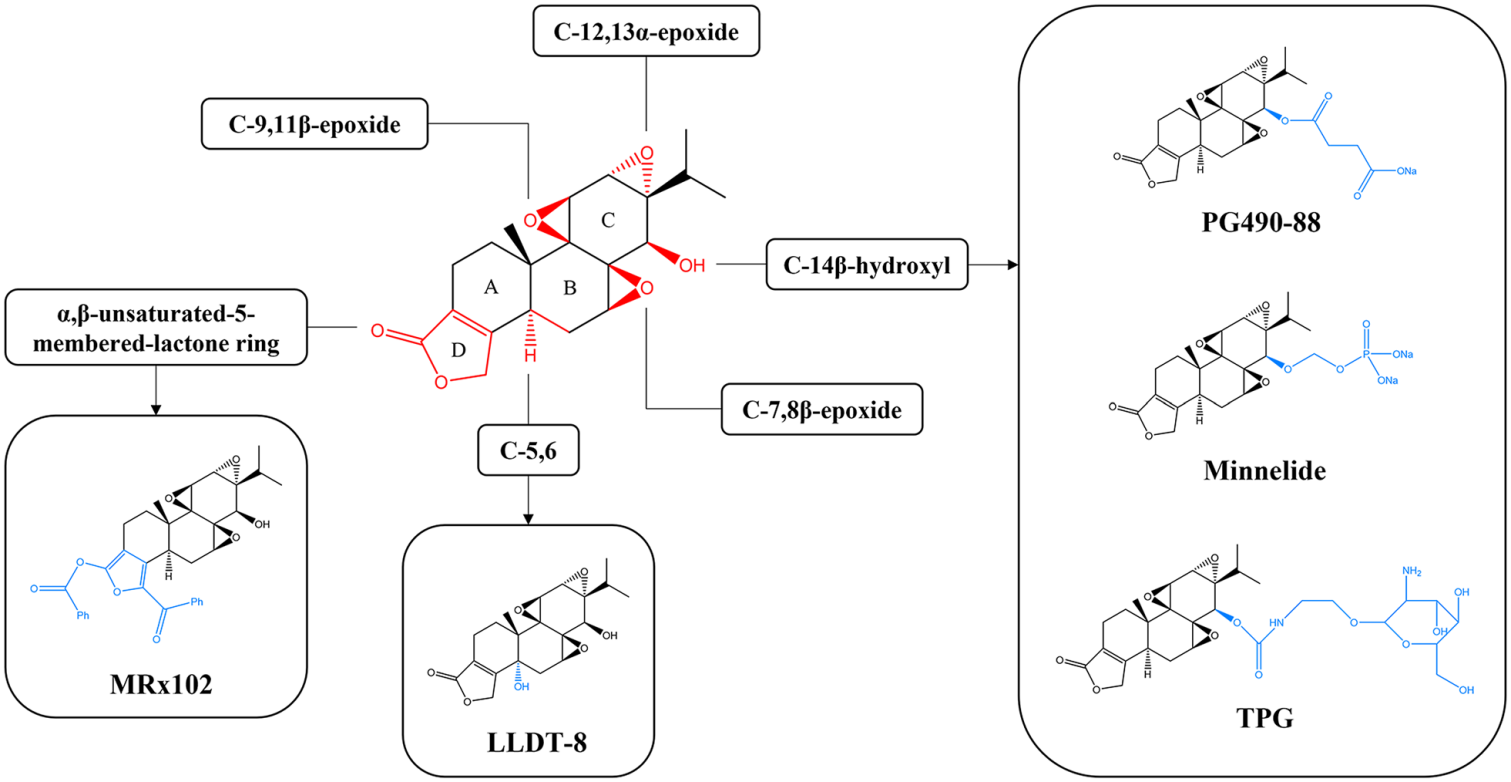
SARs of triptolide and chemical structure of representative triptolide derivatives

Many studies have attempted to enhance therapeutic effect and reduce toxicity of triptolide by modifying its structure. In this process, the structure-activity relationships (SARs) of triptolide are summarized [11, 113]: (1) proper modification of C-14β-hydroxyl can improve its anticancer activity, water solubility, target selectivity, and reduce toxicity; (2) the three epoxides are essential to its biological activities; (3) the unsaturated lactone ring (D-ring) is associated with its biological activities but can be replaced with other ring system; (4) introduction of suitable C-5,6 functional groups may retain immunosuppressive and anticancer activity with reduced toxicity. According to these SARs, a variety of triptolide derivatives have been developed and even advanced in clinical evaluation for the treatment of autoimmune diseases and cancers, like PG490-88, Minnelide, LLDT-8 (Fig. 8; Table 2). PG490-88, 14-succinyl triptolide sodium salt, is a water-soluble prodrug metabolized to triptolide after absorption into blood. It has been elucidated as an effective immunosuppressant to prevent rejection in organ transplantation and possesses potent antitumor activity [114, 115]. PG490-88 entered into phase I clinical trials in Europe for the treatment of solid tumors in 2003, while the clinical trial was discontinued later because of lethal side-effects [116]. Minnelide, as another water-soluble prodrug of triptolide, was found to be extremely effective in pancreatic and liver cancer, and the relevant clinical trials are in progress [117]. LLDT-8, also termed (5R)-5-hydroxytriptolide, have been proven to be a potential treatment for rheumatoid arthritis, multiple sclerosis, and lupus nephritis [118–120]. More important, it showed 122-fold lower cytotoxicity in vitro and a 10-fold lower acute toxicity in vivo compared to triptolide, with comparable anti-inflammatory and immunosuppressive activity [121]. MRx102, a prodrug of 19-benzoyl triptolide, showed more effective and safe in acute myeloid leukemia patient cells, which is worthy of further study in clinical trials [122].

A common and effective strategy to reduce drug toxicity is targeted delivery, which enables the drug to specifically accumulate at lesion site. The glucose-triptolide conjugates can target tumor cells that overexpress glucose transporters, thus showing higher cytotoxicity to tumor cells than normal cells [123, 124]. Similarly, triptolide-glucosamine conjugate (TPG) has been developed to target kidney, because 2-glucosamine was identified as a ligand of megalin (a receptor expressed in the renal tubule epithelium) and could significantly improve the drug uptake in kidney [125, 126]. TPG could selectively accumulate in kidney and protected renal function from acute ischemia/reperfusion injury in rats, suggesting that TPG may be a potential clinical drug for immunological renal diseases [127].

**Table 2:** Clinical evaluation of triptolide derivatives

Derivatives	Disease	Dosage regimen	Safety	Status	References
PG490-88	Advanced solid tumors	0.5–18 mg/m^2^, infusion for 2 times every 3 weeks (days 1 and 8)	Anaemia, fatigue, nausea, vomiting, diarrhoea and constipation; two cases of death at the dose of 12 and 18 mg/m^2^	Phase I: suspended	[116]
	Refractory or relapsing acute leukemia	0.15–13 mg/m^2^, infusion for 5 consecutive days every 15 days	Dose-limited cerebellar toxicity	Phase I: completed	[128]
LLDT-8	Rheumatoid arthritis	0.25–1.0 mg/day, 24 weeks	Reversible leukopenia, hematologic toxicity, and upper respiratory tract infection	Phase II: completed	[129]
	HIV-associated chronic immune activation	N/A	N/A	Phase I: ongoing	[113]
Minnelide	Pancreatic cancer	0.67 mg/m^2^, infusion for 21 consecutive days every 28 days	Reversible leukopenia, neutropenia, and cerebellar toxicities	Phase II: completed	[130]
	Advanced solid tumors	Minnelide™ Capsules, oral administration for 21 consecutive days every 28 days	N/A	Phase I: ongoing	[131]

### Novel drug delivery systems

To find innovative ways for administering triptolide and alleviating its disadvantages, various novel drug delivery systems with controlled release and targeted drug delivery have been developed (shown in Table 3), such as microemulsions, lipid-based nanoparticles, and polymeric nanoparticles.

#### Microemulsions

Microemulsions are defined as a system of water, oil and surfactant which is a single optically isotropic and thermodynamically stable liquid solution [132]. Microemulsions have been found as an effective vehicle of the solubilization of certain drugs, which can also provide prolonged release of the drug and prevent irritation despite the toxicity of the drug [133]. Xu et al. [134] prepared triptolide-loaded hydrogel-thickened microemulsion (TP-MTH), which possessed good anti-inflammatory and analgesic activities by transdermal administration. The results showed no obvious local and systemic toxicities of TP-MTH at high toxic doses in different animals, including rabbits, mice and beagle dogs. And only mild reversible skin irritation signs were observed on the skin of rabbits and guinea pigs. Chen et al. [135] developed another microemulsion-based hydrogel transdermal delivery system for triptolide to avoid its strong gastrointestinal toxicity, which had no irritation on intact skin after a single application or multiple applications.

#### Lipid-based nanoparticles

Lipid-based nanoparticles, including liposomes, solid lipid nanoparticles (SLNs), nanostructured lipid carriers (NLCs) and lyotropic liquid crystals (LLCs), bear the advantage of being the least toxic for in vivo applications [136].

Among various lipid-based formulations, liposomes, which primarily consist of phospholipids, have been approved as carriers for delivering several pharmaceutical drugs by FDA. Chen et al. [137] reported a triptolide-loaded liposome hydrogel patch (TP-LHP), which provided a more stable and long-term release of triptolide compared with intragastric administration. Before administration, they used a micro-needle array to pierce the skin and form conduits, consequently promoting transdermal absorption of TP-LHP. This transdermal delivery system had significant efficacy in CIA model, which could reduce the incidence and severity of gastrointestinal reactions.

SLNs since the early 1990s have potential attraction and market value as drug-delivery systems due to natural composition and scaled-up synthesis process. The structure of SLNs is composed of a solid lipid core, which have high resistance to drug degradation and produce sustained release when administered via gastrointestinal tract [138, 139]. Xue et al. [140] compared plasma concentration and tissue distribution of triptolide-loaded solid lipid nanoparticles (TP-SLNs) and free triptolide after a single intragastrical administration to male rats, and compared with triptolide group, TP-SLNs administration reduced fluctuations in drug concentrations with sustained release and had lower concentrations in testicular tissue with decreased reproductive toxicity. Besides, Zhang et al. [110] discovered that the application of SLNs alleviated the irritation in rat stomach tissues induced by triptolide, which could be attributed to the decrease of direct contact between drugs and mucosal surface. A carrageenan-induced rat paw edema experiment indicated that TP-SLNs administration could increase anti-inflammatory activity of triptolide and had a protective effect against triptolide-induced oxidative stress and hepatotoxicity [141]. However, a major disadvantage of SLNs is undesired drug expulsion due to ongoing crystallisation or transformation of the solid lipid during production and storage [142].

NLCs were developed as the second generation of SLNs in the late 1990s, which have imperfect crystal or amorphous lipid matrices, resulting in increased drug loading and reduced drug expulsion. Zhang et al. [110] also developed triptolide-loaded NLCs (TP-NLCs) by microemulsion technique, which manifested better sustained release and more effective resistance to triptolide toxicity compared to TP-SLNs. Except for oral administration, NLCs have been mostly researched for transdermal drug delivery system. Gu et al. [143] demonstrated that TP-NLCs could effectively penetrate into skin for alleviating knee joint swelling and inhibiting inflammatory infiltration in RA rat model.

LLCs are unique liquid crystalline phases based on self-assembly of amphiphilic lipids in an aqueous environment [144]. They are broadly classified into three categories according to their internal structures: lamellar phase (L_α_), cubic phase (V_2_) and hexagonal phase (H_2_). Their structures and chemical properties of V_2_ and H_2_ phases are similar to those of cell membranes, allowing drugs to penetrate stratum corneum for transdermal delivery [145]. Shan et al. [146] reported that the IC_50_ of triptolide-loaded V_2_ group and H_2_ group in HaCaT cells were respectively 158 and 23 times that of free triptolide group, indicating their excellent anti-arthritic effects with low skin irritation.

#### Polymeric nanoparticles

**Fig. 9:**
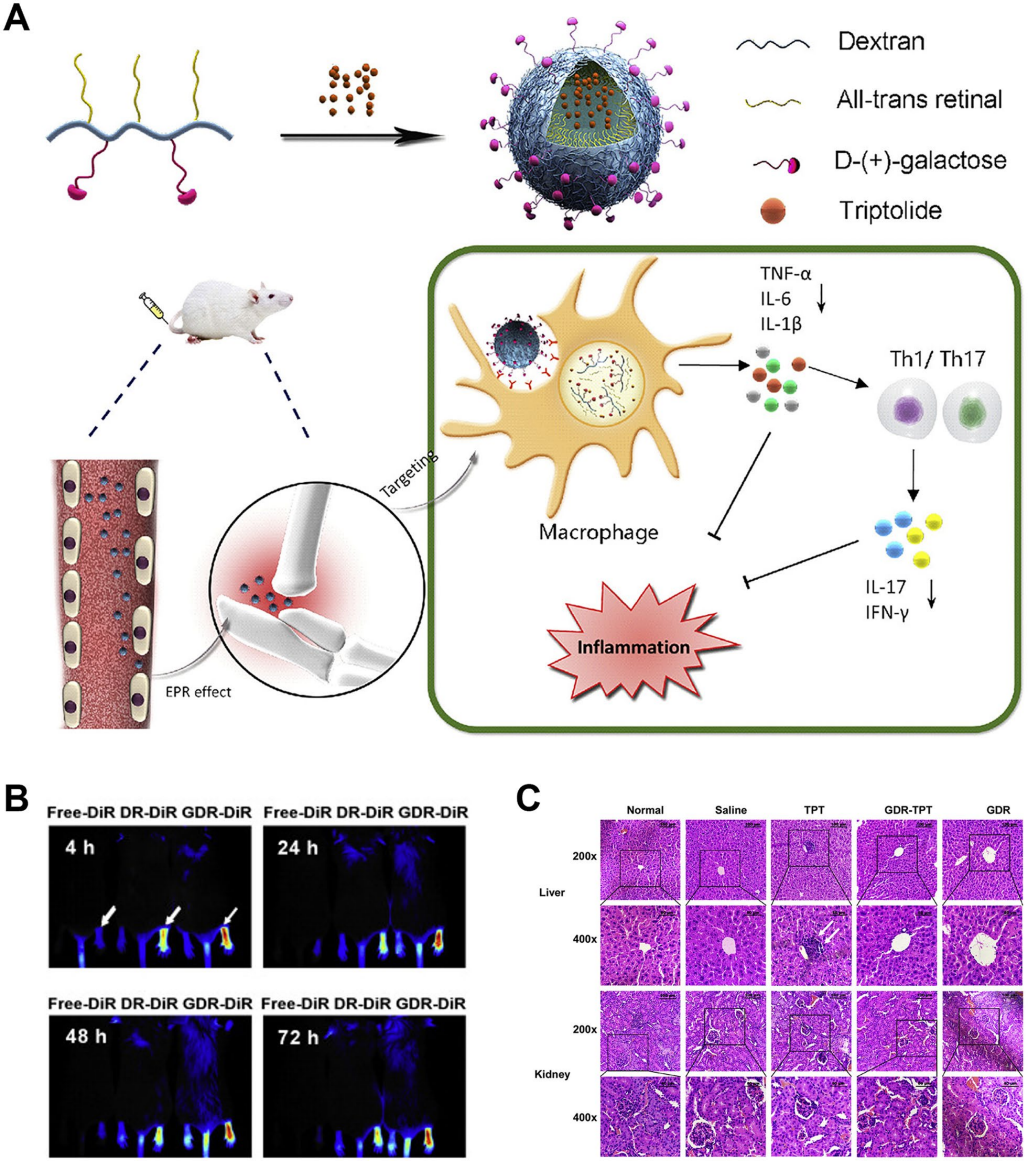
The inflammation-targeted pH-sensitive GDR-TPT not only itself has anti-inflammatory property, but also promotes drug releasing at the intracellular regions of inflamed site, thereby enhancing anti-arthritic effect and improving drug safety. **A** Scheme of GDR-TPT nanoparticle. **B** The distribution of DR and GDR from 4 to 72 h after i.v. injection. **C** Histologic results of representative liver and kidney tissues from mice with different treatment. (Reprinted with permission from [150], Copyright 2019 Elsevier B.V.)

Polymeric nanoparticles are promising drug delivery carriers because of their adjustable functions by using different polymers. Many studies have proved that various polymer carriers can reduce toxicity and guarantee the efficacy of triptolide [85, 147, 148]. Zhang et al. [149] developed a novel nano-drug carrier system (PAT) by wrapping triptolide in poly-γ-glutamic acid-grafted di-tert-butyl L-aspartate hydrochloride. They indicated that PAT could accumulate in the inflammatory joints of TNFα-Tg mice by EPR effect, with decreased death rate and toxicity at liver and spleen induced by triptolide. Furthermore, Li et al. [150] encapsulated triptolide in amphiphilic pH-sensitive galactosyl dextran-retinal (GDR), which preferentially accumulated in the inflamed joints through active targeting to galactose receptor in activated macrophages after injection into CIA mice (Fig. 9). Meanwhile, retinoic acid, the oxidized product of all-trans-retinal, possesses anti-inflammatory effect and thus synergy with triptolide. This research showed that triptolide-loaded GDR (GDR-TPT) administration could more effectively inhibit inflammatory infiltration, alleviate cartilage damage, and reduce systemic toxicity.

**Table 3:** Novel delivery systems of triptolide

Delivery system	Excipients	Administration route	Animals	Dose of triptolide	Advantages	References
Hydrogel-thickened microemulsion	Carbomer 940, isopropyl myristate, Tween 80, propylene glycol, triethanolamine, menthol and water	Transdermal administration	New Zealand rabbits	1.2 mg/kg (acute toxicity study), 0.06–0.54 mg/kg (long-term toxicity study)	No obvious toxicities were observed in a series of toxicity tests, only mild reversible skin irritation signs were observed on the skin of animals	[134]
			Kunming mice and beagle dogs	0.03–0.27 mg/kg		
			English guinea pigs	0.006 mg/kg (3 × 3 cm^2^ skin)		
Microemulsion-based hydrogel	Poloxamer 407, oleic acid, Gemseal 40, Labrasol, Tween 80, ethanol and water	Transdermal administration	Rabbits	0.24 mg (3 × 3 cm^2^ skin)	It afforded a better sustained release profile and strong permeability with low irritation when compared to microemulsions	[135]
Liposome hydrogel patch	Egg lecithin, cholesterol, Viscomate NP-700, glycine aluminum, polyvinylpyrrolidone K-90, glycerin, tartaric acid and water	Transdermal administration (after treated with microneedles)	Male SD rats	1.6, 10, 20, 40 mg/kg	It provided a more stable and long-term release of triptolide compared with intragastric administration and had significant efficacy in CIA model	[137]
Solid lipid nanoparticles	Glyceryl monostearate, soybean phospholipid, acetone, Poloxamer 188, Tween 80 and water	Intragastrical administration	Male SD rats	0.45 mg/kg	SLNs had a protective effect against triptolide-induced male reproductive toxicity due to lower concentrations in testicular tissue	[140]
Solid lipid nanoparticles	Polyoxyl 40 hydrogenated castor oil, glyceryl behenate, diethylene glycol monoethyl ether, egg lecithin and water	Intragastrical administration	Male SD rats	1.0 mg/kg	SLNs alleviated the irritation in rat stomach tissues induced by triptolide, which could be attributed to reduced lipid peroxidation levels and inflammation of the stomach mucosa	[110]
Solid lipid nanoparticles	Tristearin glyceride, Poloxamine 908, soybean lecithin and water	Intragastrical administration	Male Wistar rats and male Kunming mice	0.2, 0.4 mg/kg	SLNs increased the anti-inflammatory activity of triptolide and reduced triptolide-induced hepatotoxicity	[141]
Nanostructured lipid carriers	Compritol 888 ATO, Capryol 90, Tween 80, Transcutol HP, soybean oil and water	Transdermal administration	Male SD rats	9.3 mg/kg	NLCs could effectively penetrate into skin for alleviating knee joint swelling and inhibiting inflammatory infiltration in RA rat model.	[143]
Lyotropic liquid crystals	Phytantriol, carbitol, vitamin E acetate and water	Transdermal administration	SD rats	0.08 mg/kg	Triptolide-loaded cubic and hexagonal liquid crystals presented excellent anti-arthritic effects with no obvious toxicity	[146]
Polymeric micelles	Methoxypolyethylene glycol–poly(D,L-lactic acid)-block copolymer	Intravenous administration	Kunming mice	0.51–1.25 mg/kg	Its acute and subacute toxicities were slighter than free triptolide owing to the sustained release characteristics and anti-lipid oxidative damage	[85]
			Wistar rats	0.1, 0.3 mg/kg		
Polymeric vesicles	Poly-γ-glutamic acid-grafted l-phenylalanine ethylester copolymer	Intravenous administration	C57/B6 mice	0.5 mg/kg	It increased the survival rate of mice and reduced the damage of free TP on the liver, kidney, and spleen	[147]
Polymeric nanoparticles	Poly-γ-glutamic acid-grafted di-tert-butyl L-aspartate hydrochloride	Intravenous administration	C57BL/6 mice	0.15 mg/kg	It could accumulate in the inflammatory joints of TNFα-Tg mice by EPR effect, with decreased death rate and toxicity at the liver and spleen induced by triptolide	[149]
Polymeric nanoparticles	Galactosyl-dextran-retinal conjugates	Intravenous administration	Male Balb/c mice	0.04 mg/kg	It preferentially accumulated in the inflamed joints through active targeting in CIA mice, thus reducing systemic toxicity	[150]

### Combination pharmacotherapy

In clinic, combination treatment of multiple drugs that interact with different disease targets is a good strategy to alleviate drug toxicity. Researchers have discovered several drugs, such as glycyrrhizin [151], silymarin [152] and Panax notoginseng [153], could achieve better efficacy and lower toxicity when used with triptolide than monotherapy. Licorice was often used in combination with TWHF for RA treatment to reduce side effects and adverse reactions induced by latter. Research suggested that glycyrrhizin, a major bioactive ingredient of Licorice, could accelerate the metabolism of triptolide by activating CYP3A and reduce blood drug concentration of triptolide, resulting in significant protection against chronic liver injury in rats [154]. Moreover, combined treatment of glycyrrhizin and triptolide could produce a synergistic effect owing to anti-inflammatory effect of glycyrrhizin.

## Conclusions and perspectives

Recently, TWHF and triptolide have attracted the attention of many scientists in different fields. Great progress has been made in the synthesis [155], pharmacological activity [156, 157], and delivery system [158] of triptolide and its derivatives. In this review, we highlight pharmacological mechanisms and systemic toxicity of triptolide in the treatment of autoimmune disease. Furthermore, we pay great attention to the strategies to reduce toxicity of triptolide, aiming to provide a bright perspective for its clinical translation and put forward some guidance for further studies.

With an increasing incidence of autoimmune disease worldwide, there is an urgent requirement for effective therapeutic agents with a favorable cost-benefit ratio. As the main active ingredient from traditional Chinese medicine TWHF, triptolide has been testified to have excellent immunosuppressant and anti-inflammatory effects, making it a promising drug for the therapy of autoimmune disease. A number of preclinical studies scientifically explain its action mechanisms in rheumatoid arthritis, ankylosing spondylitis, systemic lupus erythematosus, psoriasis, inflammatory bowel disease, and multiple sclerosis, which are attributed to the regulation of various immune cells and cytokines to a large extent. Efficacy of triptolide on autoimmune disease is also related to specific cells with abnormal proliferation, such as FLSs in rheumatoid arthritis and keratinocytes in psoriasis. The traditional usage of TWHF is usually for the treatment of arthralgia and sore and ulcer of skin for a long time in clinic, respectively, and the syndromes of arthralgia and sore and ulcer of skin belong to the scopes of rheumatoid arthritis and psoriasis in Western Medicine. Therefore, our summarized above mechanisms can partly explain the reason why traditional Chinese Medicine doctor use TWHF to treat these syndromes.

Despite the great therapeutic potential, the serious toxic and side effects of triptolide during treatment cannot be ignored. In vivo experiments on different animals showed that triptolide had dose- and time-dependent toxicity to liver, kidney, reproductive system, heart, spleen, lung and gastrointestinal tract. A clinical trial also demonstrated that triptolide induced serious toxicity in patients with rheumatoid arthritis, thus causing a 47% withdrawal rate. Compared with oral or intravenous administration, transdermal administration is generally considered to be capable of reducing systemic toxicity. However, our previous work showed that topical delivery of a low amount of triptolide (0.0004%, 0.08 mg/kg) hydrogel can cause obvious toxicity to psoriatic mice skin, and even induce death of mice. CFDA once approved an ointment containing triptolide for the treatment of psoriasis, but it gradually withdrew from the market due to serious toxic and side effects. Moreover, the toxic mechanism of triptolide has not been investigated clearly, which needs further study urgently.

In order to reduce triptolide toxicity, strategies relying on chemical structural modification, novel drug delivery systems, and combination pharmacotherapy are employed by researchers. Chemical structural modification has the advantages of short development cycle, low cost, and low market risk. Although a large number of derivatives have been synthesized, most of them have been eliminated because of poor absorption or undesired distribution. Only a few derivatives of triptolide have entered phase I/II clinical trials, while several clinical trials were terminated due to serious side effects and even fatal events. Triptolide-glucosamine conjugates appear to be feasible for targeted therapy of immunological renal diseases, but further clinical verification is necessary. In addition to the derivatives obtained by structural modification, it is worth considering to search other bioactive components with enhanced efficacy and lower toxicity in TWHF. For novel drug delivery systems, controlled release and targeted drug delivery carriers are becoming more and more attractive. It is a potential exploration field to develop specific triptolide drug delivery systems according to different microenvironments of autoimmune diseases. Furthermore, current delivery systems of triptolide are mostly focused on cancer therapy, and investigations to treat autoimmune diseases are still scarce. Triptolide is often used in combination with other herbs in traditional Chinese Medicine for centuries, while little research has been performed on the mechanism of drug-drug interactions in vivo. Therefore, more effort should be put on the application of triptolide in autoimmune disease to promote its clinical transformation. Despite the existing obstacles, triptolide is still attractive and inspiring as a potential therapeutic for autoimmune disease.

## Data Availability

Not applicable.

## Abbreviations

AIA: Adjuvant-induced arthritis
AKT: Protein kinase B
ALT: Alanine transaminase
AS: Ankylosing spondylitis
AST: Aspartate transaminase
BMPs: Bone morphogenetic proteins
BUN: Blood urea nitrogen
cAMP: Cyclic adenosine monophosphate
CC: Cysteine-cysteine
CCL5: Cysteine-cysteine chemokine ligand 5
CCR5: Cysteine-cysteine chemokine receptor 5
CD: Crohn’s disease
CIA: Collagen-induced arthritis
CNS: Central nervous system
COX: Cyclooxygenase
Cr: Creatinine
CXC: Cysteine-X-cysteine
CYP450: Cytochromes P450
DCs: Dendritic cells
DSS: 4,4-dimethyl-4-silapentane-1-sulfonic acid
EAE: Experimental autoimmune encephalomyelitis
FLSs: Fibroblast-like synoviocytes
FXR: Farnesoid X receptor
GDR: Galactosyl dextran-retinal
GM-CSF: Granulocyte-macrophage colony-stimulating factor
HLA: Human leukocyte antigen
IBD: Inflammatory bowel disease
IFN-γ: Interferon gamma
IL: Interleukin
JAK: Janus kinase
JNK: Jun N-terminal kinase
LLCs: Lyotropic liquid crystals
MAPK: Mitogen-activated protein kinase
MCP: Monocyte chemotactic protein
MIP: Macrophage inflammatory protein
MMPs: Matrix metalloproteinases
MS: Multiple sclerosis
NETs: Neutrophil extracellular traps
NF-κB: Nuclear factor kappa B
NLCs: Nanostructured lipid carriers
NLRP3: NLR family pyrin domain containing 3
Nrf2: Nuclear factor E2-related factor 2
OCPs: Osteoclast precursors
Oct2: Organic cation transporter 2
OLGs: Oligodendrocytes
OPCs: Oligodendrocyte precursor cells
OPG: Osteoprotegerin
PGE_2_: Prostaglandin E2
PI3K: Phosphatidylinositol 3‑kinase
PKA: Protein kinase A
PPAR: Peroxisome proliferator-activated receptor
RA: Rheumatoid arthritis
RANK: Receptor activator of nuclear factor-κB
RANKL: Receptor activator of nuclear factor-κB ligand
ROS: Reactive oxygen species
SARs: Structure-activity relationships
SLE: Systemic lupus erythematosus
SLNs: Solid lipid nanoparticles
STAT3: Signal transducer and activator of transcription 3
TCR: T cell receptor
TGF: Transforming growth factor
TJ: Tight junction
TLR: Toll-like receptor
TNF-Tg: Tumor necrosis factor-transgenic
TPG: Triptolide-glucosamine conjugate
Tregs: Regulatory T cells
TWHF: Tripterygium Wilfordii Hook F
UC: Ulcerative colitis
VEGF: Vascular endothelial growth factor
